# Gingival inflammation, enamel defects, and tooth sensitivity in children with amelogenesis imperfecta: a case-control study

**DOI:** 10.1590/1678-7757-2020-0170

**Published:** 2020-09-28

**Authors:** Camille QUANDALLE, Adrien BOILLOT, Benjamin FOURNIER, Pascal GARREC, Muriel DE LA DURE-MOLLA, KERNER Stephane

**Affiliations:** 1 Université de Paris U.F.R. of Odontology Paris France Université de Paris, U.F.R. of Odontology, Paris, France.; 2 Rothschild Hospital Reference center for Oral and Dental Rare Diseases Paris France Rothschild Hospital, AP-HP, O-Rares, Reference center for Oral and Dental Rare Diseases, Paris, France.; 3 Rothschild Hospital Department of Periodontology Paris France Rothschild Hospital, AP-HP, Department of Periodontology, Paris, France.; 4 Cordeliers Research Center Laboratory of Molecular Oral Physiopathology Paris France Cordeliers Research Center, Laboratory of Molecular Oral Physiopathology, Paris, France.; 5 Institut IMAGINE Hôpital Necker-Enfants Malades Paris France Institut IMAGINE, INSERM UMR S1163, Hôpital Necker-Enfants Malades, Paris, France.; 6 Loma Linda University School of Dentistry Department of Periodontology Loma LindaCalifornia USA Loma Linda University School of Dentistry, Department of Periodontology, Loma Linda, California, USA.

**Keywords:** Amelogenesis imperfecta, Dental enamel, Dentin sensitivity, Gingivitis, Dental plaque

## Abstract

Gingival conditions and tooth sensitivity of young patients with amelogenesis imperfecta lack in depth studies. This case-control study aimed to compare (1) the gingival inflammation, the presence of enamel defects, and tooth sensitivity in young patients with and without amelogenesis imperfecta and (2) to investigate if any difference exists between subtypes of amelogenesis imperfecta.

**Methodology:**

We compared forty-two participants with amelogenesis imperfecta with forty-two controls matched for age, gender, and the number of examined sites. Based on interview, clinical examination, and intraoral photography, we collected data on periodontal conditions, enamel defects and the presence of tooth sensitivity. Comparison tests were performed to investigate if any difference existed between cases and controls; and among cases, between the different subtypes of amelogenesis imperfecta. We performed a post-hoc analysis for any significant difference observed.

**Results:**

We observed more gingival inflammation, enamel defects and tooth sensitivity among cases (all *p*<0.05). Participants with hypocalcified amelogenesis imperfecta had more gingival inflammation, enamel defects, and tooth sensitivity than patients with the hypoplastic and hypomature subtypes (all *p*<0.05). After adjustment for dental plaque, gingival inflammation was associated with the presence of amelogenesis imperfecta (OR (95%CI) = 1.14 (1.05; 1.24). *p*<0.01).

**Conclusion:**

Gingival inflammation, enamel defect and tooth sensitivity are more frequently observed among young patients with amelogenesis imperfecta, and more specifically among children with the hypocalcified subtype.

## Introduction

Amelogenesis imperfecta (AI) is a rare genetic disease affecting enamel development and mineralization. It might be isolated or a symptom of a syndrome, and it can affect both primary and permanent teeth. Isolated AI results from mutations in specific genes (*LAMB3, ENAM, AMBN, ITGB6, AMELX, KLK4, MMP20, WDR72, ODAPH, SLC24A4, FAM83H, DLX3, ARHGAP6, LAMA3, AMTN, ACPT, GPR68, RELT, SP6*).^1-4^ AI presents three subtypes: hypoplastic (type I), hypocalcified (type II), and hypomature (type III).^5^ The prevalence of AI reaches 1/14 000 in the USA but to date, no existing epidemiologic study has been conducted in France.^6^

Clinical expression varies, affecting teeth color (from white to yellow brown), surface (smooth, rough, spotted), and hardness (from normal hardness to soft enamel). Some patients also experience sensitivity and pain. Finally, other oral anomalies can be observed, such as teeth agenesis, pulp calcifications, open bite, gingival overgrowth, and periodontal disease.^7^

Different kinds of AI exist: hypoplastic, hypomature, and hypocalcified AI. Hypoplastic AI consists of a lack of enamel in quantity, which leads to morphological anomalies detectable on X-rays. Patients feel no pain, but some thermal sensitivity can occur.^8^ Hypomature AI corresponds to a defect of protein maturation within the enamel matrix, i.e., the presence of some proteins prevents a complete enamel mineralization. The enamel lacks translucency, appears opaque and is softer than normal. On X-rays, enamel appearance is less radiopaque.^9^ Finally, Hypocalcified AI is the most severe form of AI, where enamel mineralization is not achieved. Patients encounter pain while eating, brushing, or with thermal changes. The enamel can look brown or yellowish, with both enamel and dentin sharing the same radio-opacity on X-rays.^10^

Previous case reports have documented poor plaque control, accumulation of dental calculus, and gingival inflammation among patients with AI.^7,11-13^ Nevertheless, little is known about their periodontal conditions. We need more information on the sensitivity and periodontal status of AI patients compared to patients without it.

Furthermore, no study has compared the periodontal status associated with the different AI subtypes. However, we observed in our daily clinical practice that some patients with AI have more dental plaque, calculus, and gingival inflammation than others, and may not respond to the periodontal therapy the same way.

The present study aimed to compare the gingival inflammation between children with and without AI, also comparing enamel characteristics, tooth sensitivity, and dental plaque. Finally, we investigated if any difference existed between participants with hypomature, hypocalcified and hypoplastic AI.

## Methodology

### Study population

**Figure 1:**
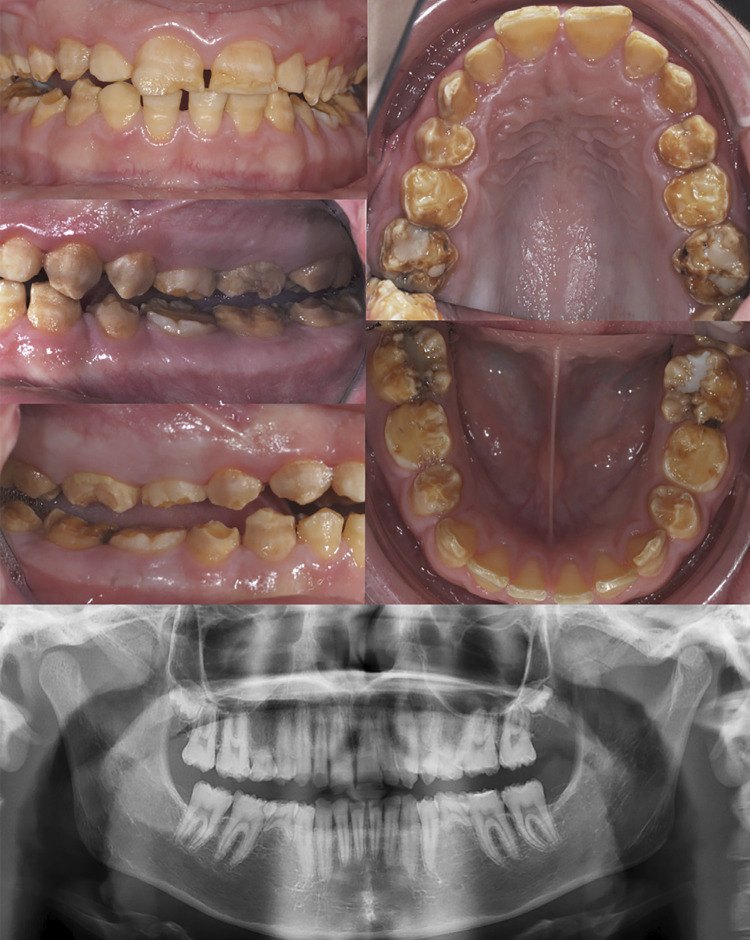
case example of clinical views and OPT of a 14 years old, male patient, with AI hypomature inflammation (PI=12%; MGI=0.19; MGI≥2 =0).

Between 2006 and 2016, we examined consecutive patients referred to the Reference Centre for Oral and Dental Rare Diseases, O-Rares, Rothschild Hospital, AP-HP (Paris, France), recording clinical and demographic data. The clinical examination was performed during the first visit, and 5 intra-oral photographs (1 front view, 2 lateral views with or without mirror and 2 occlusal views with a mirror) (Figure 1) and extra-oral photographs were taken. Evaluation included untreated restorative patients. All participants who were diagnosed with isolated and syndromic AI were included. Participants with syndromes that could affect the periodontal status such as epidermolysis bullosa were excluded. Based on dental examination, we categorized the participants into three groups: hypomature, hypocalcified, and hypoplastic AI according to criteria reported in the literature.^14^ Two independent clinicians (CQ, MM) worked on diagnosis, calling for a third practitioner (SK) in case of disagreement. As manual dexterity, and consequently the quality of tooth brushing, may vary with age, we age-matched participants across the three groups. Since both plaque and gingival index values depend on the number of scored tooth surfaces, we also matched participants for the number of sites examined.

During the same period, controls without AI who started an orthodontic treatment were included and matched with cases for age, gender, and the number of sites examined.

For retrospective data in practice surveys, French legislation only requires ensuring the protection of personal data.^15^The data file was then submitted and approved by the “Commission Nationale Informatique et Liberté” (CNIL # 2048817 03-30-2017).

### Enamel characteristics

Based on clinical examination and intraoral photography, we recorded and qualitatively evaluated the presence of enamel defects.

### Periodontal conditions

Based exclusively on intra-oral photographs, we evaluated 3 values in buccal and 3 values in lingual/palatal (mesial, centre and distal) for the plaque index and the gingival index. After anonymizing the patients, we projected the intra-oral views on a full screen for evaluation. We modified the O’Leary plaque index (PI) to calculate the participants’ oral hygiene.^16^ From the photograph, we calculated the percentage of site with visible plaque, without plaque disclosing. Gingival inflammation was assessed using the Modified Gingival Index (MGI),^17^ adapted from Löe’s (1976) Gingival Index (GI)^18^. A previous study concluded that the MGI correlated significantly with the GI.^19^ MGI is more conducive with photographic examination since it requires no probing to assess the degree of inflammation. By analogy with the GI, scores 1 and 2, both of which describe mild gingivitis, were merged to obtain MGI’s four-class index: 0: absence of inflammation; 1: mild inflammation or slight changes in color and texture; 2: moderate, bright surface inflammation, erythema, oedema and/or hypertrophy of marginal or papillary gingiva; 3: severe inflammation, erythema, oedema and/or marginal gingival hypertrophy of the unit or spontaneous bleeding, papillary, congestion or ulceration. We also calculated the percentage of sites with moderate or severe inflammation (MGI≥2).

We randomly selected fifteen participants for the calibration procedure. The periodontal examiner (CQ) was calibrated to a standard examiner (SK), and kappa coefficients for inter-examiner reproducibility for PI (PI=0 *versus* PI=1) and GI (GI<0 *versus* GI≥0, and GI<2 *versus* GI≥2) were 0.66 (95%CI: 0.61-0.72), 0.70 (0.65-0.75), and 0.87 (0.80-0.93), respectively. Using the same pictures, plaque and gingival inflammation assessments were repeated one week later and the kappa coefficients for intra-examiner reproducibility were 0.74 (0.69-0.79), 0.75 (0.71-0.80), and 0.72 (0.62-0.81), respectively.

### Definition of other covariates

We registered gender and age at first visit and assessed tooth sensitivity with the question “Do you feel pain when you eat, drink or brush your teeth?” (yes or no).

### Statistical analyses

Based on data from a previous study on the longevity of dental restorations in young patients with and without amelogenesis imperfecta,^20^ the mean percentage of sites with gingival inflammation was 26.9±24.6 in the amelogenesis imperfecta group and 12.8±14.8 in the control group. With 33 participants in each group, we considered possible to detect a mean difference of at least 14% with a standard deviation of 20% between the two groups. This estimate was based on a two-tailed test of matched pairs conducted at the 5% level of significance with a statistical power of 80%.

We compared included and excluded participants using Wilcoxon and Fisher’s exact tests.

First, we compared controls and cases using McNemar’s test and pairwise t-test. Then, we used Friedman and Cochran’s Q tests to determine differences between the three clinical subtypes. We performed a post-hoc analysis for any significant difference observed.

We used Wilcoxon tests to compare the mean plaque index between participants with and without tooth sensitivity, and participants with and without enamel defect. We plotted and quantified the linear correlation between mean plaque index and mean gingival inflammation by using the Pearson correlation coefficient. Finally, we ran a multivariate model to determine whether mean gingival inflammation was associated with the presence of amelogenesis imperfecta after adjustment for mean plaque index.

We considered statistically significant a *p* value less than 0.05. We performed all statistical analyses using R software (version 2.14.0, the R Core Development team, 2010).

## Results

**Figure 2:**
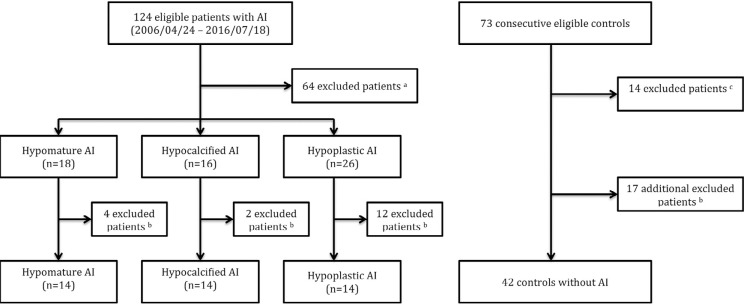
Flowchart of the study sample selection

**Figure 3:**
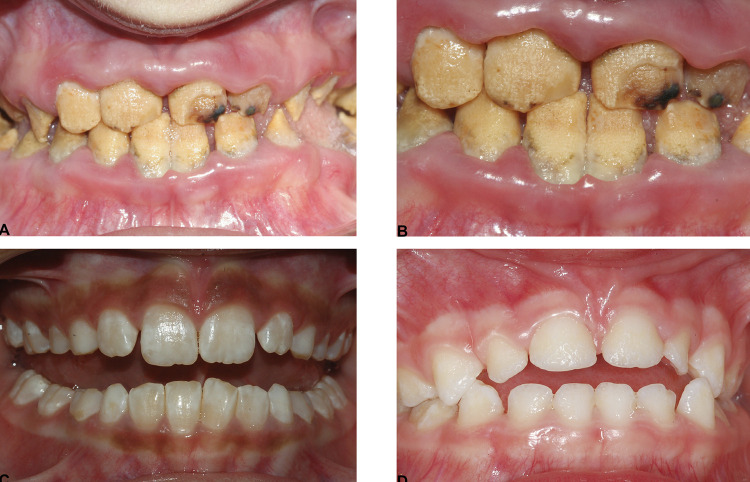
A: Clinical view of a 9 years old, male patient, with hypocalcified AI. Enamel is of normal thickness but is hypocalcified and soft. The patient presents plaque on every visible surface, associated with a severe gingival inflammation (PI=78%; MGI=1.67; MGI≥2=44%). B: same patient with higher magnification. C: Clinical view of a 9 years old, female patient, with hypomature AI. Enamel is of normal thickness, but it is mottled and softer than normal. The patient does not present visible plaque nor gingival inflammation (PI=3%; MGI=0.12; MGI≥2=0%). D: Clinical view of a 5 years old, female patient, with hypoplastic AI. The enamel is thin and pitted. The patient presents a small amount of plaque some in mesial and distal surfaces and a mild gingival inflammation (PI=7%; MGI=0.18; MGI≥2=0%)

Among the 124 eligible young patients with AI, we excluded 64 because of missing data or syndrome affecting the periodontal status, and 18 during matching. Thus, the study included 42 participants with hypomature (n=14), hypocalcified (n=14), and hypoplastic (n=14) AI as cases and, consequently, 42 matched controls (Figure 2-3).

We found no significant difference between included and excluded cases for the AI subtypes (*p*=0.05). However, excluded patients were more likely to have hypoplastic AI (66.7% *versus* 33.3%), whereas few excluded patients presented the hypocalcified form (11.1% *versus* 33.3%). Mean MGI was lower among excluded patients (0.2±0.6 *versus* 0.5±0.5. *p*<0.01). The percentage of sites with moderate and severe inflammation was also lower among excluded subjects (6.7±23.5 *versus* 11.9±16.7. *p*<0.01). We observed no significant difference between included and excluded subjects for enamel characteristics, tooth sensitivity, and enamel defect (Supplemental table 1).

**Supplemental table 1:** Characteristics of included (n=42) and excluded (n=18) cases

	Included patients	Excluded patients	*p*
Subtypes of AI			
*Hypomature*	14 (33.3)	4 (22.2)	0.05
*Hypomineralized*	14 (33.3)	2 (11.1)	
*Hypoplastic*	14 (33.3)	12 (66.7)	
Age	10.9±5.7	11.8±8.5 (n=15)	0.98
Enamel colour			
*Normal*	3 (7.1)	2 (11.1)	0.45
*White*	8 (19.1)	6 (33.3)	
*Brown*	7 (16.7)	1 (5.6)	
*Yellow*	24 (57.1)	9 (50.0)	
Enamel surface texture			
*Smooth*	18 (42.9)	8 (47.1)	0.79
*Spotted*	5 (11.9)	3 (17.6)	
*Rough*	19 (45.2)	6 (35.3)	
Tooth sensitivity (Yes)	21 (63.6)^(a)^	6 (54.5)^(b)^	0.72
Enamel defect (Yes)	31 (73.8)	11 (64.7)^(c)^	0.53
Examined sites (n)	86.4±24.3	90.5±45.2	0.49
PI (%)	29.0±28.6	13.4±22.6	0.05
Mean MGI	0.5±0.5	0.2±0.6	<0.01
Sites with MGI≥2 (%)	11.9±16.7	6.7±23.5	<0.01

Wilcoxon test and Fisher’s exact tests. (a): missing data for 9 included patients. (b): missing data for 7 excluded patients. (c): missing data for 1 excluded patient.

Due to matching, no significant difference existed for age, gender, and the number of examined sites between cases and controls, (Table 1) and between the three clinical subtypes of amelogenesis imperfecta (Table 2).

**Table 1:** Characteristics of included patients

	Controls	Cases	*p*
Age (years)*	10.9±5.2	10.9±5.7	0.98
Gender [% male (n)]	52.4 (22)	52.4 (22)	1
Tooth sensitivity (1) [% positive (n)]	4.8 (2)	63.6 (21)	0.02
Enamel defect [% positive (n)]	4.8 (2)	73.8 (31)	0.34
PI (%)*	11.4±9.5	29.1±28.7	<0.01
Mean MGI*	0.1±0.1	0.5±0.5	<0.01
Sites with MGI≥2 (%)*	0.5±1.2	11.9±16.7	<0.01

McNemar's test and pairwise t-test. (1) Nine cases have missing data for tooth sensitivity. *Mean±SD.

**Table 2:** Characteristics of included patients by subtype of AI

	Subtypes of AI			*p*		
	Hypomature	Hypocalcified	Hypoplastic			
	(n=14)	(n=14)	(n=14)			
Age (years)*	11.0±6.0	10.5±4,7	11.1±6.8	0.66		
Tooth sensitivity(1) [n(% positive)]	7 (63.6)	12 (92.3)	2 (22.2)	0.30^a^	0.02^b^	0.59^c^
Enamel defect [n(% positive)]	6 (42.9)	14 (100)	11 (78.6)	<0.01		
Examined sites (n)*	89.8±27.0	82.4±23.2	86.9±23.8	0.51		
PI (%)*	12.3±11.0	61.6±22.9	13.1±14.8	<0.01		
Mean MGI*	0.2±0.3	1.0±0.4	0.3±0.3	<0.01		
Sites with MGI≥2 (%)*	5.7±9.7	26.2±20.5	4.0±5.8	<0.01		

Friedman and Cochran’s Q tests. (1) Missing data for 3 patients with hypomature AI, for 1 patient with hypocalcified AI, and for 5 patients with hypoplastic AI. Fisher’s exact test was used for 2x2 comparisons (a) hypomature *versus* hypocalcified, (b) hypocalcified *versus* hypoplastic, (c) hypomature *versus* hypoplastic. *Mean±SD.

### Periodontal conditions

Regarding periodontal status, mean plaque index was 11.4 (Range: 0.8 - 38.4) among controls and 29.1 (0 - 93.7) among cases (*p*<0.01). Mean gingival index and the percentage of sites with moderate and severe gingival inflammation were higher among cases (respectively 0.5 (0 - 1.7) vs 0.1 (0 - 0.4) and 11.9 (0 - 65.3) *vs* 0.5 (0 - 5.6), all *p*<0.01) (Table 1). Cases with the hypocalcified subtype, when compare with the two other clinical subtypes of AI, showed worse periodontal conditions. We found no significant difference between patients with hypomature and hypoplastic AI for all three periodontal parameters (Tables 2-3)

### Tooth sensitivity and enamel defects

Only two controls showed both tooth sensitivity and enamel defects, whereas 63.6% of cases had tooth sensitivity and 73.8% had enamel defects (all *p*<0.05) (Table 1). All participants with the hypocalcified subtype had enamel defects, with 92.3% of them reporting tooth sensitivity. Tooth sensitivity was also prevalent among children with the hypomature subtype, whereas enamel defect was more prevalent among participants with the hypoplastic subtype (Table 2). Participants with the hypocalcified subtype had more enamel defects than participants with the hypomature form (Table 3). Participants with the hypocalcified subtype reported more tooth sensitivity than participants with the hypoplastic form (Table 3).

**Table 3:** Post-hoc pairwise comparisons: dental plaque and gingival inflammation (p values)

hypomature	hypocalcified	hypomature	
	*versus*	*versus*	*versus*
	hypocalcified	hypoplastic	hypoplastic
PI (%)	<0.01	<0.01	0.84
Mean MGI	<0.01	<0.01	0.98
Sites with MGI≥2 (%)	0.01	<0.01	0.84

### Plaque index, enamel defect, and tooth sensitivity

**Figure 4:**
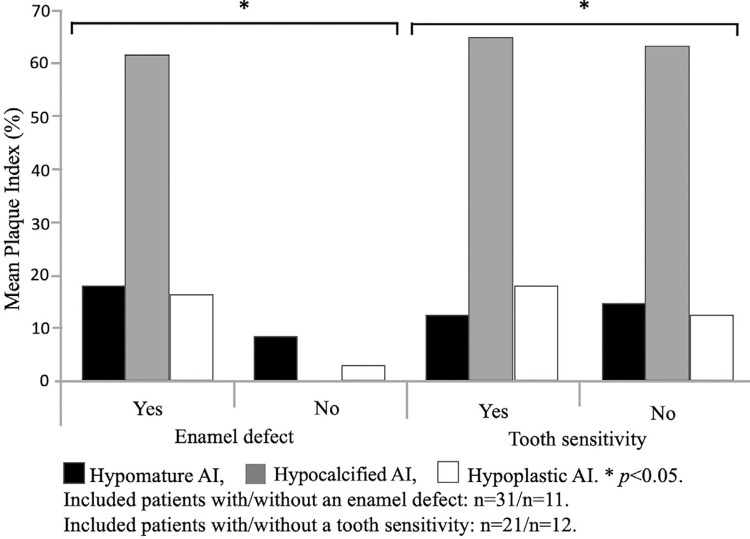
Plaque index and presence of enamel defect or tooth sensitivity

Plaque index showed to be higher among participants with enamel defects and those who reported tooth sensitivity (Figure 4).

### Plaque index, gingival inflammation, and presence of AI

**Figure 5:**
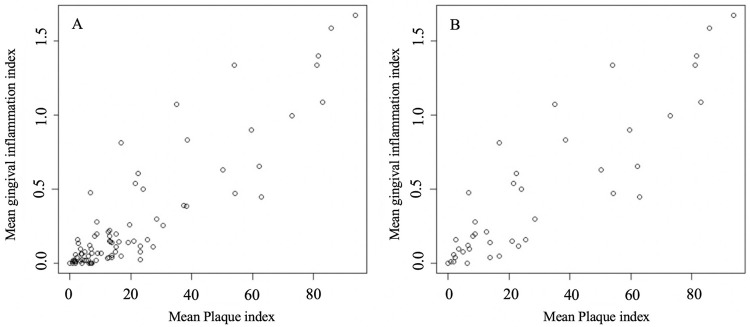
Linear correlation between mean plaque index and mean gingival inflammation among all participants (A) and among cases (B)

We observed a linear correlation between mean plaque index and mean gingival inflammation among all 84 participants (*r*=0.89) (Figure 5A), and among cases only (*r*=0.88) (Figure 5B).

After adjustment for plaque index, mean gingival inflammation was associated with the presence of AI (OR (95%CI) = 1.14 (1.05; 1.24), *p*<0.01) (Data not shown).

## Discussion

This case control study reveals that participants with AI had more tooth sensitivity and gingival inflammation than controls without AI. Among participants with AI, the hypocalcified subtype showed higher mean gingival inflammation, and more enamel defects and tooth sensitivity. The proportion of sites with moderate and severe gingival inflammation was higher among cases than controls; and more specifically among young patients with the hypocalcified subtype. We found no significant difference for periodontal conditions between participants with hypomature and hypoplastic AI. After adjustment for mean plaque index, we observed an independent association between mean gingival inflammation and the presence of AI.

A previous retrospective study compared oral health conditions and the longevity of dental restorations between patients with AI and controls. Patients with AI had more sites with bleeding on probing than controls. The study made no distinction between the different subtypes of AI.^20^ Another retrospective study compared the oral health status between participants with hypocalcified (n=5) and hypoplastic AI (n=10). The authors observed higher values for plaque and bleeding indexes, and for the percentage of sites with probing depth more than 3mm, among participants with hypocalcified AI.^11^ A cross-sectional study found similar results, with worse periodontal conditions among participants with hypocalcified AI than among those with the hypoplastic subtype.^12^ Neither of these two studies included participants with hypomature AI. To our knowledge, only one study compared periodontal status among the three subtypes of AI. In this case series, gingival index was higher among participants with hypocalcified AI. But the study included only 12 participants, including two with hypocalcified AI, and two with the hypomature subtype.^13^ In the present study, we confirm that patients with hypocalcified AI present the worst periodontal conditions.

Enamel acts as a physical barrier because of the hardness and the highly mineralized nature of this tissue. Among patients with hypocalcified AI, the enamel shows normal thickness, but mineralization is defective. Consequently, the tissue does not play its protective role, resulting in tooth sensitivity.^21^ From a biological standpoint, enamel defects observed among patients with hypocalcified AI become local risk factors for bacterial adhesion and plaque colonization.^22,23^ In the present study, we observed a significant association between the presence of dental plaque and tooth sensitivity (Figure 4). From a behavioural standpoint, we hypothesized that, among patients with hypocalcified AI, tooth sensitivity due to enamel defects results in poor plaque control and, consequently, in gingival inflammation.

The study has several limitations. First, due to the very young age of several participants, we chose to assess plaque index and gingival inflammation by using intra-oral photographs and not clinically. Photography and image analyses are frequently used in periodontology to evaluate plaque index and/or gingival inflammation, by different techniques.^24-31^ The quantitative evaluation of dental plaque requires the use of plaque disclosure; color photographs of the disclosed plaques are sensitive and reliable.^25^ Dental plaque can be evaluated by using a quantitative index, or by assessing the covering area surface. In the present study, we conducted the evaluation of dental plaque on all visible tooth surfaces with a “yes or no” index to limit the risk of error due to the absence of discoloration. The use of this type of index is more reliable than quantitative indexes when using classical cameras. The present study observed a mean plaque index among controls of 12.3%, while previous studies conducted among young European patients have described a plaque index of roughly 30%.^26,27^ The lower mean plaque index observed in the present study may be due to the dichotomic assessment of dental plaque. Evaluation by image analysis allows for reproducible comparison of changes in gingival color and/or volume. These techniques can be used to diagnose gingivitis, but also to monitor the evolution of these variables over time.^28^ Although the color of healthy gingiva may vary between people, the use of digital gingival color measurement is simple, reliable and reproducible.^29-31^ Moreover, dichotomous diagnosis based on redness is more reproducible than swelling.^30^

Secondly, no information regarding social classes, occupations, and education levels of parents as a proxy measure for socioeconomic status featured in the analysis. Socioeconomic status may impact oral hygiene habits and the frequency of dental visits, which impact periodontal conditions. Thirdly, no genetic data was available for these patients. Thus, we cannot discuss the measures with the genotypes. Finally, because of the low prevalence of AI, the study included only 42 cases.

## Conclusion

Patients with AI present more gingival inflammation and tooth sensitivity than patients without AI. Among patients with AI, oral conditions were worse in the hypocalcified subtype than in hypomature or hypoplastic AI. Most of the sample were children, and thus these conclusions might need confirmation with adults.
